# Technologies as Support Tools for Persons with Autistic Spectrum Disorder: A Systematic Review

**DOI:** 10.3390/ijerph110807767

**Published:** 2014-08-04

**Authors:** Nuria Aresti-Bartolome, Begonya Garcia-Zapirain

**Affiliations:** DeustoTech-LIFE Unit, DeustoTech Institute of Technology, University of Deusto, Avda. Universidades 24, Bilbao 48007, Spain; E-Mail: mbgarciazapi@deusto.es

**Keywords:** ASD, tools for therapy, robots, telehealth systems, dedicated applications, virtual reality applications

## Abstract

This study analyzes the technologies most widely used to work on areas affected by the Autistic Spectrum Disorder (ASD). Technologies can focus on the strengths and weaknesses of this disorder as they make it possible to create controlled environments, reducing the anxiety produced by real social situations. Extensive research has proven the efficiency of technologies as support tools for therapy and their acceptation by ASD sufferers and the people who are with them on a daily basis. This article is organized by the types of systems developed: virtual reality applications, telehealth systems, social robots and dedicated applications, all of which are classified by the areas they center on: communication, social learning and imitation skills and other ASD-associated conditions. 40.5% of the research conducted is found to be focused on communication as opposed to 37.8% focused on learning and social imitation skills and 21.6% which underlines problems associated with this disorder. Although most of the studies reveal how useful these tools are in therapy, they are generic tools for ASD sufferers in general, which means there is a lack of personalised tools to meet each person’s needs.

## 1. Introduction

According to the Diagnostic and Statistical Manual of Mental Disorders (DSM-V), Autistic Spectrum Disorder is a group of alterations which appear between 12 and 14 months of age and is characterized by social interaction and communication problems and repetitive behavior [1,2].

Studies show that there has been an increase in ASD in recent years. Several authors have attributed this to a greater awareness [3], recognition, and diagnosis of the disorder and the fact that less severe cases being included in the spectrum [4]; in addition to continuous changes in the definition of ASD [5]. However, there is no consensus on the prevalence of ASD because there are many autism-related syndromes [6], due to diagnoses based on clinical criteria [3].

Nevertheless, studies show that more cases of autism have been detected. There are publications that show that 1 child out of every 150 or 110 out of every 10,000 children are affected in 2009 [7], or studies conducted on pre-school age children in Spain that show a prevalence of 8.1% and 11.7% [7]. This indicates that the prevalence of this disorder has risen 78% since 2002 [8,9]. The study presented by Mayada *et al.* [10] confirms an average estimated prevalence of 62 per 10,000. Recent results were presented in March 2014 by Centers for Disease Control and Prevention (CDC) which show that about 1 in 68 was identified with ASD in USA [11].

Due to the increase in diagnosed cases of ASD, software and hardware dedicated to persons with autism have been developed for several decades. These solutions reinforce ASD sufferers’ strong points and work on their weaknesses, helping them to increase their vocabulary and communication [12] skills [13,14]. These studies mostly concentrate on one of the core areas affected by ASD, communication (the worse their communication problems, the more severe the symptoms of ASD are [15]).

Tortosa [16] states that Information and Communication Technologies (ICTs) can compensate and support education of students with special needs, and particularly people with ASD. ICTs make it possible to create controllable predictable environments; they offer multisensory stimulation, which is normally visual; they foster or make it possible to work autonomously and develop the capacity for self-control and are highly motivating and reinforcing [17], encouraging attention and lessening the frustration that may arise from making mistakes [18].

However, there are authors who maintain that “computers make persons with autism more autistic”. In other words, they believe the use of technology can further isolate ASD sufferers who have problems in social relationships or can cause them to have obsessive compulsive behavior [19]. However, when used correctly, ICTs may work to improve social interaction due to their multiple uses and options [16,20].

With new technologies, one are able to get a closer look at the lonely world of autism, prompting a better understanding of ASD sufferers’ mental state and helping them to develop skills which would not be possible without the subject-technology interaction. ICTs work to penetrate the isolation of people with autism and bring them out of the “world apart” in which they live [21,22].

The use of these technologies has been so successful that research using ICTs has increased from one publication in 1970 to more than 38 a year at the present time [20], appearing not only in impact journals targeting the social field [23], but also in the technical field [24]. In addition to scientific research, there are a large number of blogs where family members post how their children interact with them.

Specialist literature contains numerous reviews of studies including technology as support and help tools, proving the benefits of their use. Examples include the work by Ploog *et al.* [20], Wang *et al.* [25] or Scassellati *et al.* [26]. However, they offer little information on the most recent studies and concentrate mainly on the areas they target without any division by type of technology or applications.

The following division was used:

- Virtual reality applications
- Dedicated applications
- Telehealth systems
- Robots

The research was further divided according to the area affected by ASD which is targeted in each study. Several article databases such as Scopus, IEEE Xplore, ACM Digital Library or Web of Knowledge were consulted to carry out the review, but as most of the articles indexed in these data bases are also contained in Web of Knowledge, this database has been chosen to make the review.

The following inclusion criteria chosen for this study:

- Articles published between 2004–2014.
- Articles indexed in Web of Knowledge.
- Studies which work on affected area of ASD.
- Studies which incorporate technologies such as virtual reality, robots, telehealth systems or dedicated applications to detect, diagnose or improve the ASD.

This review is therefore organised in the following sections: firstly, the mixed reality applications for persons with ASD are analysed. Secondly, the dedicated applications, and thirdly the leading telehealth systems. Fourthly, the studies conducted with robots. The final section offers a discussion on the analysed studies and our conclusions.

## 2. Mixed Reality Applications

The term “mixed reality” has been used for years to refer to virtual reality and augmented reality technologies. Mixed reality makes it possible to create and develop worlds in which real and computer-created elements are merged [27,28].

Due to the advantages of using this technology to create controlled and real environments, there is research that proves how it can be used in a controlled manner as a useful efficient support tool in areas such as, for instance, health [29,30,31], defense [32]. In ASD, mixed reality can help us to understand how children with autism are challenged by a sensory overload and aversion to a variety of visual and tactile stimuli [33].

**Figure 1:**
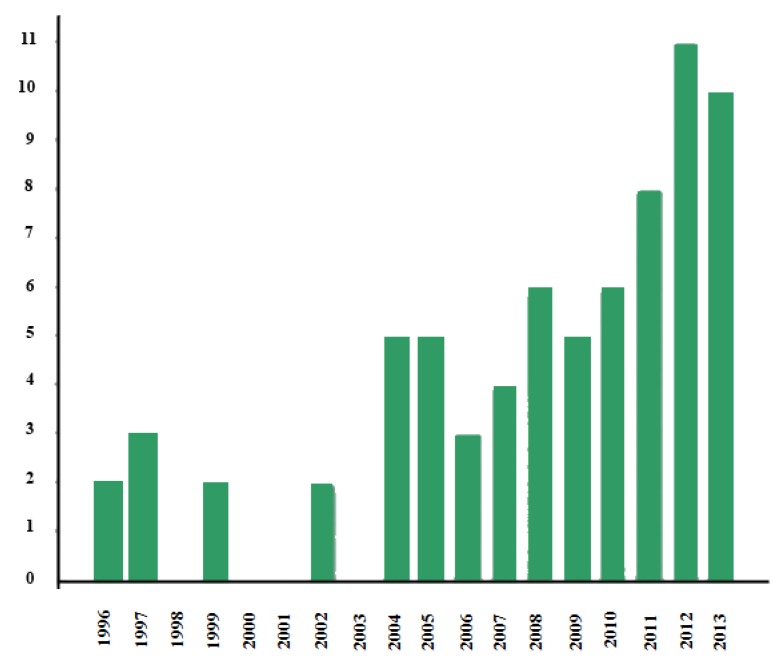
Graph of studies on virtual reality and autism.

The Web of Knowledge was searched with the keywords “autism” and “virtual reality” to find the leading studies on this technology. As can be seen in Figure 1, the first studies go back to 1996, with others being carried out from time to time until 2004 when research on the subject increased.

The virtual reality applications developed for ASD can be classified by the areas they focus on. The following categories were established for this review: Communication and interaction, social learning and imitation skills and other associated conditions. 

### 2.1. Communication and Interaction

Ke *et al.* [34], developed virtual environments engaging the participants with autism in social situations and different exercises. The first task was to recognise the body language and facial expressions of avatars, the second was to communicate with them in a school cafeteria and finally, interact with them at a birthday party. The researchers carried out an analysis based on observing the participants and completing questionnaires. They obtained positive results as the children demonstrated that communication and interaction during the intervention had increased as did their communicative competences following the tasks.

Brigadoon [35] gives us another example of virtual worlds with a program based on interaction of people with mental disorders. The aim is to stimulate people with Asperger syndrome or autism to learn to socialise, providing them with an environment where they can interact with each other. As Brigadoon was a pilot online community for people dealing with Asperger’s Syndrome and Autism developed by BrainTalk Communities, there are no published scientific results.

In 2006, Parson *et al.* [36] studied the behaviour of two adolescents with ASD in two virtual environments, a café and a bus. In this study, the authors proved that the adolescents significantly interpreted the scenes and appreciated the opportunities to maintain a dialogue and respond correctly although they continued to show repetitive behaviour and interpret the situations literally. Mitchell *et al.* followed this same line of research [37] created a virtual coffee shop. 6 adolescents with ASD were shown 3 sets of videos of real situations taking place in coffee shops and cafés followed by the virtual environment. They had to say where they had decided to sit and why. This answer was analyzed and coded by 10 evaluators. Half of the participants were shown the virtual environment between the first and second set of videos and it was shown to the second half during the second and third set of videos. The researchers found that there were cases of significant improvement, directly related to the time spent in the virtual world when deciding and explaining where they chose to sit. Stickland *et al.* [38] developed a tool called JobTIPS which made it possible to teach job interview skills to people with high-functioning autism. They used visual support aids, videos, guides on the theory of mind and virtual worlds where they practised these skills. Twenty two young people took part in the experiment to check the effectiveness of the program. Half of the young people completed sessions with the programme while the other half that formed the control did not use the system. Following the experiment, the participants who had used the programmer showed significantly better verbal skills during the interview than the control group.

### 2.2. Social Learning and Imitation Skills

Researchers Josman *et al.* [39] developed a safe environment using virtual reality technology which enabled persons with ASD to learn how to cross the street. Six children with ASD formed the experimental group and six children with neurotypical development formed the control group. The researchers concluded that persons with ASD learned the skills needed to make the right decisions when crossing the street in a virtual environment and thus, the knowledge acquired could be applied to real situations.

Virtual environments have also been studied to help learn skills such as playing. Herrera *et al.* [40] conducted two case studies on children with autism in which they evaluated this skill with virtual environments. The findings showed improvement in play skills following the intervention.

Fabri *et al.* [41] centered their research on how persons with autism interact with avatars capable of facial expressions showing emotions (happiness, sadness, anger and fear). In the first stage of the experiment, the participants (34 young people diagnosed with ASD, average age of 9.96) had to choose the emotion the avatar was expressing from a list. In the second stage, the avatar appeared in a social environment and the participants had to interpret what emotion the scene involved. In the third and final stage, the participants had to select what caused the emotion the avatar was expressing from a list of events or situations. The authors checked that 30 of the participants understood the emotions of the avatars and used them appropriately. However, the other four participants, who were in the group that described themselves as having severe autism, had a real difficulty in understanding the emotional representation of the avatars.

Ehrlich *et al.* [42] developed a 3D virtual world called Animated Visual Supports for Social Skills (AViSSS) at the University of Kansas in 2008. This system enabled people with Asperger syndrome to work on social skills using different environments and situations shown on the platform. Participants had to choose how to behave or select objects. This platform afforded them the opportunity to practice different social situations without the tension or anxiety involved in the real world. During the initial tests, the authors concluded that the students with ASD did not respond well to the virtual avatar, virtual teacher specifically, due to the fact that, they appeared to perceive teachers as being uninterested, impatient to deal with them.

### 2.3. Other Conditions

This technology has also been used to motivate people with autism to do exercise. Finkelstein *et al.* [43] developed a game called Astrojumper. Users had to dodge virtual objects that appeared on the screen. Herrera *et al.* [44] carried out a pilot study which took advantage of the game provided by Kinect. They developed a set of educational games in which children did exercise (using their bodies as the control mechanism) and which also made them more aware of their own bodies. Studies have also been conducted to examine how people with ASD interact with the real world. Fornasari *et al.* [45] created an urban environment where they compared the behavior of neurotypical children and children with ASD. It consisted of two exercises. In the first one, the children explored the environment freely and in the second, they went round the environment to fulfil the goals set. The researchers found that there were no differences in behaviour between the two groups in the second task. However, in the first task, children with ASD took less time to explore the environment than the neurotypical children did, with significant behavioral differences between the two groups.

### 2.4. Conclusions

Good results have been obtained by using virtual reality applications as therapeutic tools, thus helping people with autism to recognize emotions and improve their social and cognitive skills [46].

Virtual reality makes it possible to create safe environments where they can learn rules and repeat the tasks. Furthermore, interacting with avatars where social situations are replicated enables patients to work on these situations and find more flexible solutions. This means that virtual environments may be good instruments to work on social skills with ASD sufferers [47,48].

This technology makes it possible to create avatars or more real looking characters to enable participants with autism to work on facial expressions and emotions and recognise them [41,49] while also creating controlled environments to make them feel safe [50,51]. Therefore, this technology provides advantages that can be used as a support tool in therapy. Verbal and gesture-based interaction can be worked on in virtual reality or mixed reality environments, achieving effective neurorehabilitation in children [24,25]. Table 1 contains a summary of the studies analyzed.

## 3. Dedicated Applications

In this paper, technological tools targeting people with autism are called dedicated applications (virtual reality is not used). They are designed to be used on computers, tablets or mobile telephones.

Applications dedicated to people with autism are mostly support tools to facilitate or assess their skills when communicating, with a focus on social skills. This study therefore analyzes the tools found to be most significant. They are divided into the following groups: (1) Communication (2) Social learning and imitation skills (3) Other associated conditions.

**Figure 2:**
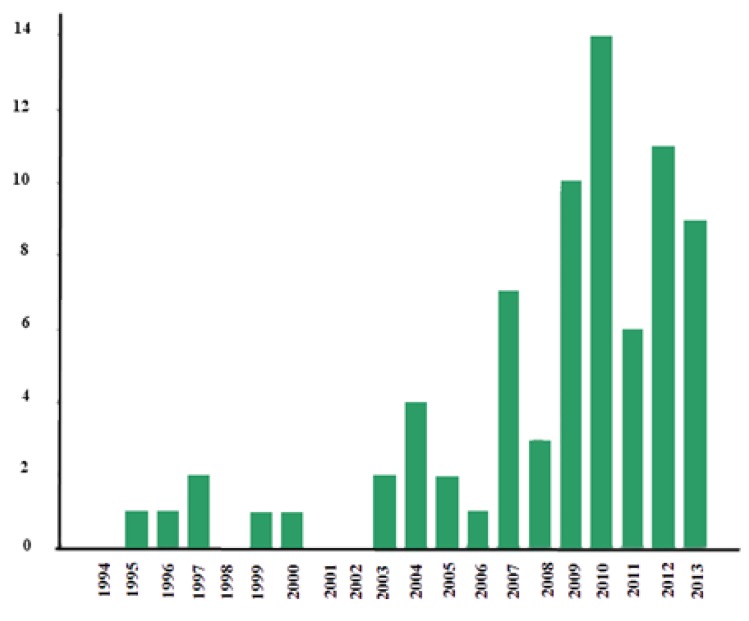
Graph of computer application and autism research.

The keywords used to search the most relevant studies on this technology in the Web of Knowledge were “autism” and “computer application”. As shown on the graph (see Figure 2), the first studies go back to 1995, with research having been conducted off and on since then. Research on the subject began to increase after 2007. 2010 was the year most research on this topic was carried out. 

**Table 1:** Studies on mixed reality systems.

Author	Year	Country	Clinical Group	Control Group	Age	Diagnosis	Area	Method	Results	Classification
Ke *et al.* [34]	2013	USA	4 Children	-	4–5	High-functioning ASD	Social interaction	Virtual-Reality + Persons	Communication and interaction during intervention increment	Social learning and skills imitation
Brigadoon [35]	2006		-	-	-	-	Social interaction	Virtual-Reality	Brigadoon. Pilot online community	Communication and interaction
Parsons *et al.* [36]	2006	UK	2 adolescents	-	-	ASD	Social Communication	Virtual environments, café and bus	Adolescent interpreted the scenes and responded correctly	Communication and interaction
Mitchell *et al.* [37]	2007	UK	6 teenagers	-	-	ASD	Communication	Virtual environments, cafe	Improvement related to time spent when they were making decisions	Communication and interaction
Stickland *et al.* [38]	2013	USA	22 teenagers	-	16–19	High-functioning ASD	Job interview skills	Virtual-reality, videos, Theory of Mind guides	Improvement on verbal skills	Communication and interaction
Josman *et al.* [39]	2008	Israel	6 children	6 children	-	ASD	Social skill: Cross the street	Virtual environment	Learning the skills needed to make right decisions	Social learning and imitation skills
Herrera *et al.* [40]	2008	Spain	2 children	-	8:6	ASD	Play skills	Virtual environment	Play skills improvement	Social learning and imitation skills
Fabri *et al.* [41]	2007	UK	34 young people	-	7–16	18 Asperger and 16 with severe autism	Social skills	Virtual avatars	88.3% of participants understood the emotions of avatars	Social learning and imitation skills
Ehrlich *et al.* [42]	2009	USA	Adolescents	-	-	ASD	Social skills	AVISS virtual environments, school, gymnasium	Participants did not respond well to the virtual avatar	Social learning and imitation skills
Finkelstein *et al.* [43]	2010	USA	-	8 people	4 (11–16 years) 2 (18–25 years) 2 (40–50 years)	Neuro-typical	Physical exercise	Astrojumper: a virtual reality game, dodge objects	Pilot study which works on physical exercise	Other conditions
Herrara *et al.* [44]	2012	Spain	-	-	-	-	Motor skills	Kinect and educational games	Pilot study which made children more aware of their own bodies	Other conditions
Fornasari *et al.* [45]	2013	Italy	16 children	16 children	-	ASD	Behavior	Urban virtual environments (1-free exploration 2-defined objects)	1° task: children with ASD took less time to explore environment than control group. 2° task: no behaviour differences	Other conditions

### 3.1. Communication

People with autism experience serious difficulties in social interaction and conversation [52], so the majority of the applications center on improving their communication. In 2008, Grynszpan *et al.* [53], developed software especially for people with ASD. It consisted of three games. These games were used to work on one of the areas affected by this disorder: communication. The software included subtitled dialogues that expressed irony, sarcasm and metaphors, in addition to faces showing emotions. Participants had to understand the situation shown and respond correctly. Ten adolescents diagnosed with ASD and 10 neurotypical adolescents took part in the study. The software was used once a week for 13 weeks. The researchers used the results obtained in the first and last sessions to evaluate each participant’s skills. The results showed that the adolescents with ASD performed poorly on rich multimedia interfaces because they lacked initiative when organising the information given in the multimodal sources.

In 2011, the Orange Foundation and the Dr. Carlos Elósegui Foundation at the Guipuzkoa Polyclinic developed software that facilitated communication for people with ASD. It is called e-Mintza and uses tactile technology and icons, symbols such as pictograms and ARASAAC graphics. E-Mintza easily adapts to users’ needs. It also fosters their automony via a personalized agenda [54]. In line with this type of systems, a communicator called Piktoplus was developed. Piktoplus is a tool based on the System of Augmentative and/or Alternative Communication (SAAC). It was designed to facilitate communication for anyone who cannot use and/or understand verbal speech. It consists of a tactile table formed by pictograms that enable the user work on: language, behavior guidelines, motricity graphs and specific cognitive areas by playing [55]. ZacPicto is another similar system. ZacPicto is tool created to help parents and professionals to work with people with autism. The programme provides a visual organizer which makes it possible to organise and structure all the activities as well as a communication space via a social network for everyone involved in caring for people with autism: parents, teachers, therapists [56,57].

Applications for tablets or PDAs are another line of research. Torii *et al.* [58,59] developed Lets Talk! in Japan. Lets Talk! is a programme for personal digital assistant (PDA) systems that help users to communicate by selecting images and sounds from the programme. The system’s effectiveness and usability was checked with a 9 year-old child with autism. After using the system, his bad behaviour improved as he learned to express his thoughts and interests appropriately with the application. In 2003, Ganz *et al.* [60] proved the efficiency of the use of tablets as communicator systems over conventional communicators. Three people with ASD took part in the study, two of whom quickly learned to use the system and stated that they preferred it over conventional systems. However, the third participant was not skilled at using it and preferred the former systems.

### 3.2. Social Learning and Imitation Skills

Research on the effectiveness of music in therapy with people suffering from ASD has been conducted since 1964 [61,62]. These studies show how therapies including music help to persons with autism to learn new concepts and skills [63,64,65]. Music has therefore been included in applications used as treatment tools. For instance, in 2009, Hoelzl *et al.* [66], developed a prototype tool to create music called “Constraint Muse” for high-functioning children with autism or Asperger’s syndrome and people suffering from Parkinson’s Disease. The system used Nintendo’s Wii control to make it easy to use [67] and create music. It also fostered collaborative play by allowing several people to create music together.

Studies were also carried out to check emotion recognition skills. Tanaka *et al.* [68] used the emotion skills battery Let’s face it! in 2012, in which they compared 68 ASD children and 66 neurotypical children as they labelled social emotions such as happiness, anger, disgust, surprise, *etc.* shown on faces. The ASD children had worse results than the control group when naming happiness, sadness, disgust and anger. They also analysed how the children examined the facts and found that the children with ASD paid more attention to the mouth than the eyes while it was the opposite in the neurotypical children.

Another study was the one conducted by Hulusic *et al.* [69], in which a framework was created to help people with autism to learn new skills. They developed four games that taught the participants pointing skills during the games. This skill is thought to be necessary to learn other new ones. The study proved the usability of the tool and obtained very positive results since the children participating were able to use it easily. The children also transferred the knowledge they acquired to other environments.

Chanchalor *et al.* [70] demonstrated that computer games which included art activities and songs improved the capacity of the five participating children to learn colors and develop their imagination after using them for 6 weeks. Thus, they confirmed that adapted tools are useful to work on and improve skills.

### 3.3. Other Associated Conditions

Skills related to play and the imaginations are also studied by using dedicated applications for persons with autism. One such example is by developing systems that include story tellers [71]. Murdock *et al.* [72] used an iPad that told stories develop communication while playing. Four small children with autism took part in the study. They played with videos featuring dolls that produced interactive dialogues and encouraged the children to participate. After using the system, the three participants managed to increase dialogue and even produce new dialogues during the game. However, one of the children showed no improvement after using the game. Another interesting study was carried out by Dillon *et al.* [73] in 2011 in which an application enabled children with autism to invent stories. Through the children’s creations, they were able to analyze writing skills and imagination in children with autism in comparison to neurotypical children. They found that children with autism and neurotypical children invented real stories and fantasies and that both groups invented more stories based on real facts than fantasy. However, in both groups the logic used was better in the fantasy stories. The researchers found that the two groups used different aspects of the application to create their stories. The neurotypical children made no errors whereas the children with autism did. This proved that this disorder affects the imagination.

Sarachan *et al.* [74] worked on the imagination via the Scratch programme. Children used it to invent their own stories and games and it enabled children with autism to develop and strengthen problem-solving capacity and creativity (areas that this disorder usually affects).

An application called ZacBrowser is also of interest. ZacBrowser is a browser developed especially for children with autism and autism spectrum disorders. It is divided into several categories (aquarium, television, games, music, stories and blackboard) and leads the child to webpages with content for children, thus avoiding the possibility of entering unsuitable pages or those that contain too many stimuli that could distort the user’s attention [75].

Computer games have also been developed which explore prosodic focus and linguistic components of spoken phrases [76]. The children listened to pairs of pre-recorded phrases whose content and intonation varied in the practice phase and then heard a recombination of them in the actual test phase. The children had to select one of the two phrases whose content and prosody varied. The researchers found that during the practice phase, the children with autism made similar selections when choosing phrases according to content or their prosodic features while the children with normal development showed a clear preference for content over prosody. However, both groups discriminated between the practice stimuli and the recombination of test stimuli.

Their capacity for expression was studied through the system designed to evaluate syntactical awareness [77]. The children learned to touch words on a screen in the correct sequence to see the corresponding animation. Although the results varied, it was found that the users lacked syntactical awareness but their command of basic syntax in the non-voice domain was higher than what they howed when speaking.

### 3.4. Conclusions

Applications of this type have been used to work on the areas affected by autism and conditions related to the autism spectrum disorder, mainly concentrating on creating applications that help persons with autism to communicate through images and sounds.

These systems are widely accepted because they are simple to use and contain very intuitive tools, since they work with everyday items. However, it is important to remark that these are pilot studies so it must still be demonstrated that users can transfer these new skills to their everyday lives.

It is therefore important to continue developing these systems and further research in the field to tackle key challenges such as communication and interaction. Including the human component in systems is considered essential. In other words, another person must take part in the system, thus obliging autism sufferers to communicate. Table 2 shows a summary of the most relevant studies on dedicated applications which are analyzed in this paper.

**Table 2:** Studies on dedicated applications.

Author	Year	Country	Clinical Group	Control Group	Age	Diagnosis	Area	Method	Results	Classification
Grynszpan *et al.* [53]	2008		10 adolescents	10 adolescents	-	ASD	Communication skills	Subtitled dialogues (irony, sarcasm and metaphoras); images of facial expression	Participants with ASD performed poorly on rich multimedia inter-faces.	Communication and interaction
Fundación orange [54]	2011	Spain	-	-	-	ASD	Communication	Emintza: Communicator using pictograms	Pilot study. Software facilitated communication	Communication and interaction
Limbika [55]	2012	Spain	-	-	-	ASD	Communication	Piktoplus: Communicator using pictograms	System which works on language, behaviour guide-lines, motricity.	Communication and interaction
Fundación orange [56,57]	2012	Spain	-	-	-	ASD	Communication	ZacPicto: Communicator using pictograms	Tool which helped parents.	Communication and interaction
Torii *et al.* [58,59]	2013	USA	1	-	8 years	Autism	Communication	Lets Talk!	Bad behaviour and learning to express thoughts enhancement	Communication and interaction
Ganz *et al.* [60]	2013	USA	3 children	-	3–5 years	ASD	Communication	Tablet as a communicator	2 of 3 children preferred the new system	Communication and interaction
Chancha-lor *et al.* [61]	2013	-	5 children with ASD	-	11–15 years	ASD	Abilities to learn about colors	Art activities, game and folklore on computer multimedia	Improve on learning colors and developing their imagination	Social learning and imitation skills
Hoelzl *et al.* [66]	2009	Germany	-	-	-	Asperger or Parkinson	Collaborative play and imagination	Constraint Muse: Music + Wii control	Prototype tool to create music with Nintendo Wii control	Social learning and imitation skills
Tanaka *et al.* [68]	2012	Canada	68	66 typically developing control	-	ASD	Social deficits (facial emotions)	Let’s Face It! Emotion Skills Battery	Children with ASD paid more attention to the mouth than eyes	Social learning and imitation skills
Hulusic *et al.* [69]	2012	USA	4 children with ASD	-	-	ASD	Teaching basic skills and concepts	Four games for developing matching, pointing out and labeling skills	The children transferred the knowledge they acquired to other environments	Social learning and imitation skills
Chanchalor *et al*. [70]	2013		5 children with ASD		11-15 years	ASD	Social deficits	Activities in the computer media	Improvement on abilities to learn about colors	Social learning and imitation skills
Murdock *et al.* [72]	2013	USA	4 children	-	49–52 months	ASD	Communication	iPad play story	3 of 4 participants increased dialogue and produced new dialogues	Other associated conditions
Dillon *et al.* [73]	2011	UK	10 children	10 children	Average 8.96 and 8.60	ASD (High-functioning)	Imagination	Application based on creating stories	Both groups invented more stories based on real facts than fantasy, but the clinical group made mistakes	Other associated conditions
Sarachan *et al.* [74]	2012	USA	-	-	-	ASD	Creativity	Scratch: Create stories and games	Developing and strengthening problem-solving capacity and creativity	Other associated conditions
Ploog *et al.* [76]	2009	USA	9 children	9 children	-	ASD (Low-functioning)	Prosodic focus and linguistic components	Computer game	Children with ASD made similar selections according to content or prosodic features. Control group showed preference for content over prosody	Other associated conditions
McGonigle-Chalmers *et al.* [77]	2013	Scotland, UK	9	-	-	Low-functioning autism	Language	Learning computer game: 3task (2 words Noun Verb, 3 words-Noun Verb Noun and 4 words-Noun Verb Preposition Noun)	Users lacked syntactical awareness	Other associated conditions
Golan *et al.* [78]	2006	UK	19 adults	24 adults		Asperger and High-functioning autism	Complex emotions in faces and voices	Interactive multimedia	Users learned to recognize a variety of complex emotions and mental states.	Social learning and skills

## 4. Telehealth Systems

There are dedicated applications to help not only persons with ASD but also their families. This is the case of telehealth systems. They enable patient-doctor information exchange without having to go to the medical facilities, reducing the costs involved [79,80]. For this reason, research on the benefits of telehealth systems cover many fields of health [81,82,83] and are directed to adults as well as children [84,85,86].

**Figure 3:**
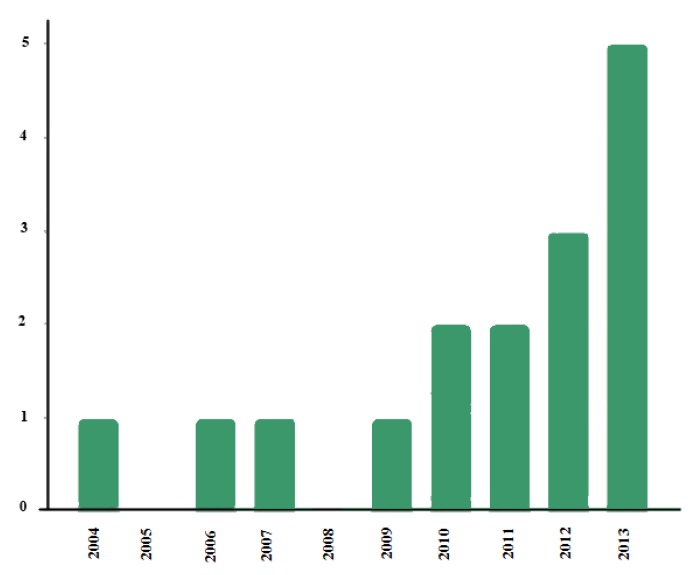
Graph of studies on telehealth and autism.

Due to the benefits these systems offer, the concept was transferred to the world of ASD and centers on helping family members caring for people with autism. The keywords for the search on the Web of Knowledge were “autism” and “telehealth”. As shown in the graph (see Figure 3), this technology was not included in the field of ASD until 2004 although it has increased, with five impact studies published in 2013. 

These studies mainly focus on helping family members of people with ASD to gain new knowledge about the disorder and as a tool to obtain information when diagnosing or determining treatment. Therefore, the following division was made: (1) telehealth systems for use by family members and (2) telehealth for diagnosis and treatment of ASD.

### 4.1. Telehealth for Use by Family Members

One example is that developed by Baharav *et al.* [87], which aimed to inform family members how to continue their children’s treatment in the home. They therefore compared the telehealth system developed to the traditional clinical model (speech and language therapy sessions) by using it once a week. The parents of two children diagnosed with autism took part in the study and stated that the telehealth system was as useful as traditional therapies, enabling them to continue with their children’s treatment from the home.

In 2012, researchers Wacker *et al.* obtained a similar result in their study. Wacker *et al.* developed a system which enabled family members to receive information on functional communication to identify and reduce behaviour problems [88,89]. The same year, Vismara *et al.* conducted a study which showed that telehealth systems made it possible for family members of persons with ASD to learn early intervention techniques and put them into practice in the day to day [90]. Following this line of research, Kobak *et al.* [91] assessed a web-based system that gave parents access to information about how to improve interaction with their family members with ASD. It was based on evidence-based practice and use of this system to maximize learning. They evaluated the effectiveness of tutorials and the family members’ knowledge before and after the experiment. They found that family members’ knowledge about how to communicate with their children improved and they also felt capable of using these communication techniques on a daily basis.

### 4.2. Telehealth for Diagnosis or Treatment of ASD

Researchers Oberleitner *et al.* conducted numerous studies on telehealth systems for use in diagnosis and treatment [92,93,94]. They developed a “tele-behavior” health system that enabled family members and carers to compile accounts of the spontaneous behavior of persons with ASD which was later analyzed by specialists [92]. This behavior was captured by using video technology, which made diagnosis quicker and more accurate [93,94].

Another telehealth system which has given optimal results in this field is the one developed by Parmanto *et al.* [95] in 2013. The system included videoconferences, recordings, images and videos, *etc.* facilitating face to face assessment of persons with ASD without having to go to therapies or clinics, *etc.*

Resee *et al.* [96] found a gap between the first suspicion of autism and diagnosis, above all in rural environments. They therefore developed a system by which persons suspected to have autism were assessed via telehealth. The researchers assessed the items which appear in the Autism Diagnostic Observation Schedule (ADOS)—Module and the Autism Diagnostic Interview-Revised (ADI-R). Their findings showed that the reliability of the system was similar to face to face sessions.

Gorini *et al.* [97] added the efficiency of virtual environment systems to telehealth systems to improve the latter. They developed the virtual world Second life, used as a stage where different disorders such as ASD can be treated. Through virtual reality, avatars were included, which allowed users to interact and improve patient-professional interaction and communication.

### 4.3. Conclusions

This subsection analyzes the most relevant telehealth systems (see Table 3). Early intervention in autism can improve the quality of life of people diagnosed with this disorder. However, not all of them receive early intervention [91]. Parents are the first to detect any problem with their children and their effectiveness as intervention agents has been proven. However, supervision by highly qualified professionals is needed [87]. For this reason, the efforts made in the field of telemedicine for people with ASD focus on creating tools that help family members or clinicians to gain knowledge about ASD [98].

**Table 3:** Studies on Telehealth systems.

Author	Year	Country	Clinical Group	Control Group	Age	Diagnosis	Area	Method	Results	Classification
Baharav *et al.* [87]	2010	USA	Parents of 2 children with ASD	-	-	ASD	Compare a traditional model of twice-weekly speech and language therapy sessions and clinic/telepractice model	Traditional model and clinic/telepractice model	Telehealth system as useful as traditional therapies	Telehealth for use by family members
Wacker *et al.* [88,89]	2013	USA	20 young children		29–80 months	ASD	Problem behavior (conducted functional)	Information exchange	Receiving information on functional communication to identify and reduce behavior problems	Telehealth for use by family members
Vismara *et al.* [90]	2012	USA	9 families with ASD			ASD	Language and imitation skills	Helping parents understand and use early intervention practices	Systems facilitated learn early intervention techniques	Telehealth for family members
Kobak [91]	2011	USA	23 parents with a child between 18 months and 6 years with ASD	-	-	ASD	Parents’ knowledge	System usability scale(SUS) and user satisfaction questionnaire (USQ)	Communication with their children improvement	Telehealth for use by family members
Oberleitner [92]	2004	USA	-	-	-	ASD	Facilitate the capturing and communication of spontaneous patient behaviors	Video Technology	Communication enhancement	Telehealth for the diagnosis or treatment of ASD
Oberleitner [93]	2006	USA	-	-	-	ASD	Diagnosis and treatment of autism	Video Technology	Diagnosis quicker and more accurate	Telehealth for the diagnosis or treatment of ASD
Oberleitner [94]	2007	USA	-	-	-	ASD	Child’s behaviors	Video-capture technology	Diagnosis quicker and more accurate	Telehealth for the diagnosis or treatment of ASD
Parmanto [95]	2013	USA	-	-	-	ASD	Diagnosis or treatment of adults with ASD	Videoconferencing, stimuli presentation, recording, image and video presentation, and electronic assessment scoring	Facilitating face to face assessment	Telehealth for diagnosis or treatment of ASD
Reese *et al.* [96]	2013	USA	10 children	11	3–5 years	ASD	Clinicians’ ability to assess autism via telemedicine	Videoconferencing	The reliability of the system was similar to face to face session	Telehealth for diagnosis or treatment of ASD
Gorini *et al.* [97]	2008	Italy	48 participants			ASD	Language skills	Telehealth system with virtual reality	Improvement on patient-professional interaction and communication	Telehealth for diagnosis or treatment of ASD

These systems have been widely accepted and received positive evaluations from families and doctors because the service is easy to use and convenient to access at any time. The systems make it possible to reduce health care costs [94,99]. However, there are gaps in these systems because they do not include tools to work on the areas affected by ASD through games and they target family members rather than people with ASD [87,100]. Nevertheless, telehealth systems are useful tools that allow for fluent communication between clinicians and family members, providing the latter and people with ASD a great deal of support.

## 5. Robots

In addition to the systems and technologies described, there are studies that analyze the behavior of people with ASD in response to robots designed to work on areas affected by this disorder.

**Figure 4:**
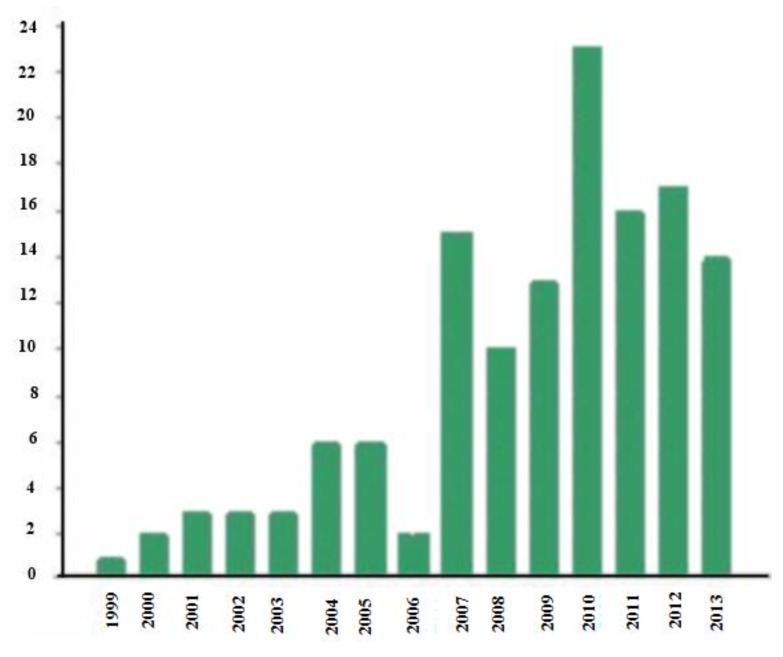
Graph of studies on robots and autism.

Robots have interesting characteristics that make them useful as tools to treat ASD [22,101,102]. Robots show predictable behavior, produce controlled social situations and interact with persons in a simple manner. This makes people with ASD feel less anxious by making social situations less complex [103,104]. The keywords used for the search on the Web of Knowledge were “autism” and “robots”. The first studies date from 1999 and have gradually increased with many being carried out in 2010 (see Figure 4). 

Like the other technologies explained above, research on the use of robots in therapy specifically focuses on social communication and social learning and imitation skills, with promising results.

### 5.1. Communication and Interaction

The literature on social communication contains different studies that aim to analyze the behavior of autistic children when interacting with robots equipped with social capacities, which are used in therapies. Huskens *et al.* [103] researched and compared the effectiveness of robots in social interventions based on applied behavior analysis. These researchers proved that intervention with robots was just as efficient as human intervention when motivating children with ASD to ask questions. Goodrich *et al.* [104] included a robot managed by the Wii control in 16 treatment sessions. The children interacted with it for 10 min. The robot consisted of a screen which showed the robot face making different expressions. The researchers analyzed the children’s behavior with the robot, including language, gestures, eye contact, imitation and demonstrations of affection. They found that the children were very motivated to interact with the robot and after the treatment with the robots, they interacted more with the clinicians than at the beginning of the study.

Kim *et al.* [22] conducted an experiment in which they found that children with ASD communicated more with adults when they played with a dinosaur robot. They ran three experiments with 23 children with ASD. The children had to interact more with one adult than another, or with a tactile screen or a dinosaur robot. Besides communicating more with the adult, they showed that children talked as much to the dinosaur robot as to the adult in charge of the session.

Although robots help people with autism to communicate, Lee *et al.* [105] conducted two studies which analyzed the social behavior of autistic children with robots. Fifteen children with ASD and robots with a “face” but which could not talk took part in the first study. In the second study, they analyzed the verbal capacity between the robot and six children with low-functioning autism comparing it to the children’s interaction with an adult. The findings of these experiments showed that, in the first case, the robots with faces foster work on social skills and facial expressions in children with autism but have no influence on the development of other skills. However, in the second case, they proved that the children interacted better with robots that could talk, following their verbal instructions and facial expressions better than with persons. Thus, robots that can talk may be an option in therapies.

However, not all the children with ASD reacted the same way to the robots. One example is the study by Tapus *et al.* [106]. They used the robot Nao which is capable of imitating the children’s movements in real time, analyzing the looks, smile, arm movements, *etc.* The study showed how two of the four children with ASD showed no change for the parameters analyzed. The other two participants showed greater eye contact with the robot than the other child and only one of the children made more spontaneous movements when interacting with the robot than with the other children.

### 5.2. Social Learning and Imitation Skills

Jordan *et al.* [107] studied the use of robots to work on attention, communication and social skills in adolescents with ASD. They recorded parameters while the participants played the card game called Face Match in different environments: with a humanoid robot, a Smart Board and the cards. The participants played for three days and the researchers recorded their behavior as they interacted with the three environments. Following the sessions, the researchers found that although there were individual behavior patterns during the three game modes, repetitive behavior was reduced when the adolescents played with the robot or the Smart Board.

Imitation is another skill that can be developed with robots. Srinivasan *et al.* [108] found that after eight sessions using robots to work on this skill, the child with ASD improved in imitation specific tasks when using the robot. Following this line of research, Srinivasan *et al.* [109] studied how children with ASD imitated a robot by making karate and dance movements. The researchers ran eight practice sessions with 15 typically developing children and four children with ASD/ADHD and eight test sessions in which they evaluated the children’s evolution. The results showed that the participants made fewer errors during the test than during the practice session, thus improving imitation-specific tasks.

However, these robots developed do not provide an individualized system for each ASD sufferer. For this reason, Bekele *et al.* [110] developed a robot with augmented vision with a camera network that obtained the head tracking in real time. The robot is capable of adapting and generating reinforcement and messages through head movements made by the person with ASD. This fosters work on social skills with each child individually.

Another capacity robots used in autism therapy have been equipped with, besides facial expressions, is the ability to tell stories in which they teach children with ASD how to act in social situations. This is the case of the robot Probo developed by Vanderborght *et al.* [111] which shows children how to react to everyday situations by saying: “Hi”, “Thanks” or “Share toys”.

### 5.3. Conclusions

Robot toys can help special needs children to work on social skills, learn new skills and discover the different game modes, in other words, show them that collaborative games also exist [110]. Thus, social robots may become very useful tools in therapy with ASD children [101,102,103,104,105,106,107,108,109,110,111,112,113].

Due to the inclusion of social robots in therapy, one has even observed how the children’s limited interests and repetitive behavior have improved. However, although robots are an effective tool, we must not forget that collaboration from people is always needed in therapy or treatment [103]. All robots do not achieve the same objective so it is interesting for therapy robots to be equipped with voice technology in order to foster social skills in persons with autism [105]. Table 4 lists the most relevant research. 

## 6. Discussion

This article presents a review of the most relevant applications and technologies developed from 2004 to 2013. Most of the results of this research, mixed reality tools, dedicated applications, telehealth systems or robots have been very positive, usually reaching the objective set for each study. All the studies show that technologies make it possible to work on the areas affected by the disorder, creating controlled environments where ASD sufferers feel safe and comfortable [105]. However, which technology is the most suitable for use in therapy? Can we conclude that these technologies really serve to teach new skills that improve these people’s quality of life?

After having analyzed the studies one by one, it is important to take a closer look at the advantages and disadvantages of these technologies as a whole and compare them to find a satisfactory answer to these questions.

**Figure 5:**
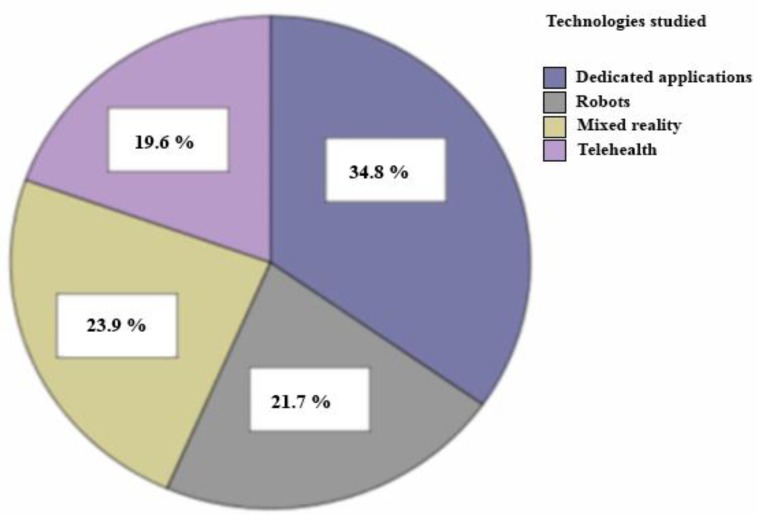
Summary of the technologies reviewed.

This study shows how research has tended to more studies on the effectiveness of applied dedications and mixed reality for this group in recent years (see Figure 5). As we have mentioned in the section on telehealth systems, this concept is very recent so there is not as much research as on the other technologies (see Figure 5). However, after having demonstrated the efficiency of these systems with other groups, they are gradually being included to help people with ASD and their families or carers [81,86].

**Table 4:** Studies on the use of robots in therapy for children with ASD.

Author	Year	Country	Sample	Control Group	Age	Diagnosis	Area	Method	Results	Classification
Kim *et al.* [22]	2013	USA	24 children	-	4–12	ASD	Social Behavior	Interaction with (1) another adult human, (2) a touchscreen computer game, and (3) a social dinosaur robot	Children talked as much to the dinosaur robot as to the adult	Social learning and imitation skills
*Huskens et al.* [103]	2012	Netherlands	6 children	-	8–14	ASD	Self-initiated questions	intervention conducted by a human or by robot	Intervention with robots was just as efficient as human intervention	Communication and interaction
Goodrich *et al.* [104]	2012	USA	2 children	-	3	ASD	Interaction	Social Robots	After the treatment with the robots, participants interacted more with the clinicians	Communication and interaction
Lee *et al.* [105]	2012	Japan	21 children	Children 6–15 years	-	ASD-Low-functioning autism	Social communication skills	1-robots with social communication skills 2-robots with verbal communication functionalities	The children interacted better with robots that could talk	Communication and interaction
Tapus *et al.* [106]	2012	France	4 children	-	-	ASD	Social skills	Nao robot (eye gaze, gaze shifting, free initiations and prompted initiations of arm movements, and smile/laughter)	2 of 4 participants showed greater eye contact with the robot than the other child	Communication and interaction
Jordan *et al.* [107]	2013	New Zealand	3 adolescents	3 adolescents	-	ASD	Attention, communication, Social skills	Memory card matching game (robot, Smart Board, playing cards)	Reduction of repetitive behavior	Communication and interaction
Srinivasan *et al.* [108,109]	2011 2013	USA	2 children	15 typically developing children	7–8 (clinical group)		USA	2 children	Imitation-specific tasks improvement.	15 typically developing children
Bekele *et al.* [110]	2013	USA	6 children	6 typically developing children	-	ASD	Deficit area of early social orienting	humanoid robot with augmented vision	Robots promoted social skills work with each child individually	Communication and interaction
Vanderborght *et al.* [111]	2012	USA	4 children	-	4–9	Austism	Social skills learning	Robot Probo (story teller)	Learning how react to everyday situations	Social learning and imitation skills

**Figure 6:**
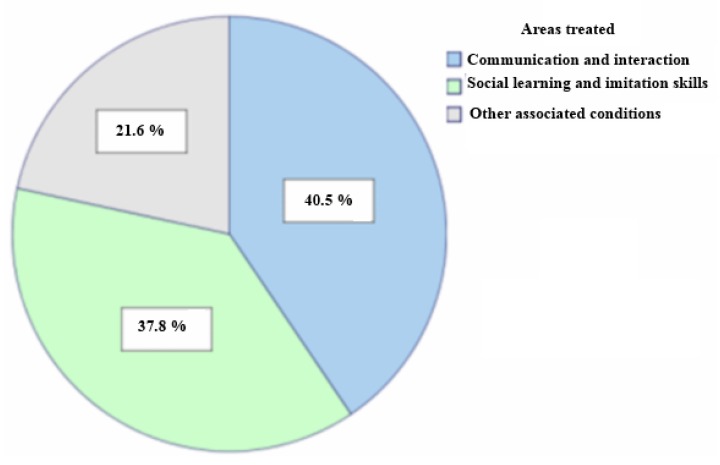
Analysis of the areas treated by using mixed reality, dedicated applications and robots.

Analysis of the studies on mixed reality, robots and dedicated applications related to the skills they focus on shows that 40.5% of this research highlights communication and interaction (see Figure 6). This is because communication and interaction is one of the core areas affected by the disorder and therefore a key aspect in therapy and the home [1,2]. In the research reviewed, all the technologies targeting communication have obtained good results and satisfactorily met the objectives set by the researchers [34,35,36,37,38,53,54,55,56,57,58,59,60,103,104,105,106].

**Figure 7:**
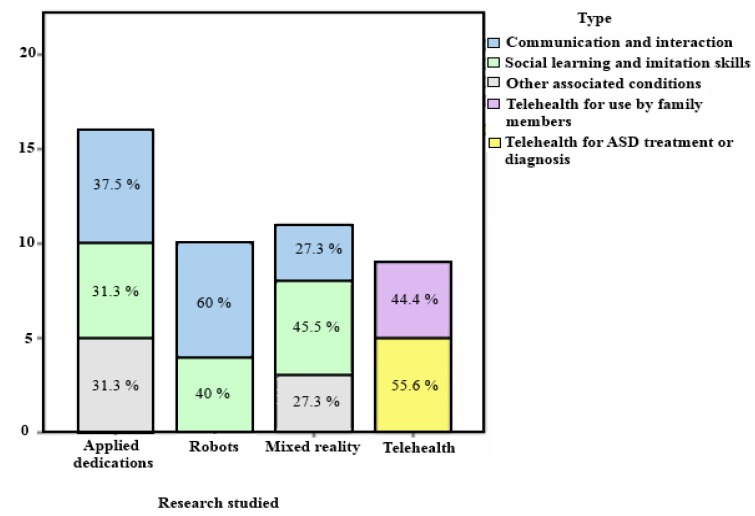
Analysis by technology and area being treated.

However, it has been observed that social robots and virtual reality are the most suitable technologies for work on communication and interaction because they focus on involving the users or participants in social situations they need to be capable of coping with. However, 37.5% of the studies on dedicated applications (see Figure 7) mainly center on providing tools that help to communicate by generating phrases with visual support aids. Put differently, users form phrases by using technological support for images, audio and texts that are reproduced when they need to communicate with other people to express their needs or feelings. However, it has not been demonstrated that they improve or learn to communicate with these applications [60].

Virtual reality makes it possible to create environments and avatars that can more realistically reflect the social situations people may be involved in and show them how they should behave in these situations. They produce situations in which the user has to communicate with other virtual components or interact with them [34,35,36,37,38]. Robots equipped with social and verbal capacities make it possible to work on robot-person interaction because they attract the users’ attention.

As for social learning and imitation skills, 37.8% of the studies analyzed focus on these areas (see Figure 6). When analysing the research by technologies, 45.5% of the mixed reality studies, 31.3% of the dedicated applications studies and 40% of the research with robots focus on this aim (see Figure 7).

The researchers conducting these studies have indicated that these technologies can also foster learning and imitation of several social skills, which is the case of mixed reality, by crossing the street or learning play skills [39]. The dedicated applications center on working on facial expressions [68], imagination [66], or learning colors [70] and robots focus on imitation [108,109].

However, these studies have their limitations as only the tools developed with robots are collaborative [112], in other words, the only tools that make it possible for more than one person to interact simultaneously. Thus, most of the research involves people with autism working individually with the tool, although there is a therapist present and directing the session.

When analyzing the results of these tools to work on problems associated with the disorder, we found that 21.6% of the studies center on these problems (see Figure 6). When checking the studies by each technology, 27.3% of the mixed reality studies and 31.3% of the dedicated applications studies center on associated conditions (see Figure 7).

Thus, we found studies focused on areas such as imagination [71], creativity or language [76] in the case of dedicated applications and motor skills and behavior in the case of mixed reality. Mixed reality is thought to be the best technology for work on motor skills because it includes hardware such as Kinect and makes the user’s own body carry out all the actions in the system.

Mixed reality systems are thought to be the best technology for work on motor skills because they include hardware such as Kinect in which the user’s body carries out all the actions in the system. In other words, it turns the user into the control mechanism without the need for an input device such as a mouse or keyboard. With the further advantage of games, users perform tasks while enjoying themselves and forget that they are exercising and thus working on motor skills [114]. However, this area has not been treated with dedicated applications because they are not active tools.

It was not possible to compare telehealth systems and robots, dedicated applications and mixed reality because they have not concentrated on the areas of communication and imitation, social learning skills or other associated conditions. This is due to the fact that telehealth systems have only recently been used to work with autism and research centers on establishing contact between family members and clinicians to show them aspects of the disorder or obtaining images of people with ASD to analyze their behavior. Although these systems have proven successful as support tools to treat different pathologies [115], facilitating person-clinician communication, there are no studies that include exercises for persons with ASD to do at home. This would make it possible to use objective variables to check ASD users’ evolution.

A comparison has been carried out on the features of these technologies in the research for which they were used. As we can observe in Table 5, key parameters were analysed: usability of the systems, if the systems are invasive, accessibility, how the data are collected, efficiency and cost.

Analysis of the use of these technologies in the research showed that they have high usability and accessibility because they are specifically designed for people with ASD so they are simple to use. Invasive techniques have not been used in the studies reviewed. They do not pose risks to users or intrude in any way [11,12,13,14,15,16,17,18,19,20,21,22,23,24,25,26,27,28,29,30,31,32,33,34,35,36,37,38,39,40,41,42,43,44,45,46,47,48,49,50,51,52,53,54,55,56,57,58,59,60,61,62,63,64,65,66,67,68,69,70,71,72,73,74,75,76,77,78,79,80,81,82,83,84,85,86,87,88,89,90,91,92,93,94,95,96,97,98,99,100,101,102,103,104,105,106,107,108,109,110,111,112]. Data collection varies depending on the platform, with the mouse input device as the most widely used. The users also used their own bodies to interact with the mixed reality system [43], and with robots and telehealth systems, the use of recordings was the most widely used to obtain information about the participants’ behavior [92,95,96]. The studies show that all the technologies are suitable for use as support instruments to work on areas affected by autism. The last parameter analyzed is related to the cost of developing the tools using the technologies reviewed. The cost is mainly related to the licenses for the libraries used. This is the case of mixed reality, where there are free or paid libraries. The dedicated applications and telehealth systems are the least expensive technologies. However, the robots can be considered the most expensive due to the material required for their manufacture.

**Table 5:** Comparison of the characteristics of the technologies.

Characteristics/Technology	Mixed Reality	Dedicated Applications	Telehealth Systems	Robots
Usability	Yes	Yes	Yes	Yes
Invasive systems	No	No	No	No
Accesibility	Yes	Yes	Yes	Yes
Data acquisition	Mouse/body movement	Mouse/tactile screen	Mouse/recordings	Observation/recordings
Effectiveness	Yes	Yes	Yes	Yes
Cost	Average	Inexpensive Inexpensive Expensive	Cost	Average

## 7. Conclusions

The conclusion reached after the analysis carried out in this study is that technology serves as a key support instrument for people with ASD, their families or professionals treating them.

Technologies can help to work on skills that ASD sufferers may not have developed because they produce repetitive controlled situations where users can exercise their strengths and weaknesses time after time, enjoying themselves and not causing tension. Thanks to the repetitive behavior of technology tools, these people do not expect any improvised social reaction like those that occur in the real world with social situations involving a large number of stimuli and variants. These environments produced by researchers are controlled to reduce the participants’ stress.

However, it is essential to ensure that the content fits the children’s ages and to set limits for the use of these technologies. Just as they can help to practice strengths and improve weaknesses in people with ASD, they can also create addiction and lead to further isolation.

In addition, mixed reality, robots and dedicated applications achieve interaction with inanimate objects whose behavior is set and predictable, which makes users feel secure and comfortable working with them. Although this is an advantage, it has the limitation of not being totally real situations with the variables involved in interaction in everyday life. For this reason, more research is needed to demonstrate how training with these technologies improves skills that are transferred to the real world, thus improving users’ quality of life.

A further limitation in these studies is the fact that the tools are developed for the entire autism spectrum. In other words, the tools work on all the users’ affected skills in a similar manner, regardless of the severity of their condition or diagnosis. For this reason, the obtained results in these studies could be altered, if the cognitive functions or language evolution are analysed. Therefore, they may not fit each individual’s needs, which could lead to a lack of interest in the system. The capacities these technologies offer may mean that it would be interesting to develop configurable systems that could be adapted to each person, thus achieving more efficient tools.

Although the studies analyzed do not include robots as tools to work on motor skills, they may be a good option because of the success shown when working on imitation. In this manner, robots could reproduce sequences of movements that users could imitate. This would make it possible to work on both imitation and motor skills.

Another remarkable aspect is that this research consists of pilot studies with a small number of participants, being mostly children, and further analyses have not been conducted following the research to check if the participants have maintained the improvement achieved during the tests. In other words, if the participants have been able to transfer the results achieved to their daily lives.

Following this analysis, it is important to conduct studies which combine various technologies in the same system to take advantage of each of them and where the main focus area is technology-ASD person-family/clinician interaction rather than technologies-person interaction. This may make it easier to transfer the skills they have worked on with the tools to daily life because persons with ASD would constantly be exposed to real social stimuli in controlled environments.

Technology can therefore give important support in therapy and diagnosis of persons with ASD and may even help to obtain objective values which enable us to understand autism a bit more and what people with autism feel in their day to day. This helps professionals to adapt therapies to each person, and families to work from their homes and gain a better understanding of their children’s behavior and needs.
